# Impact of Link Unreliability and Asymmetry on the Quality of Connectivity in Large-scale Sensor Networks

**DOI:** 10.3390/s8106674

**Published:** 2008-10-24

**Authors:** Yanjun Li, Ye-Qiong Song, René Schott, Zhi Wang, Youxian Sun

**Affiliations:** 1 State Key Laboratory of Industrial Control Technology, Zhejiang University, China / 38, Zheda Road 310027, Hangzhou, Zhejiang Province, China; E-Mails: yjli.iipc@gmail.com; 2 LORIA-Nancy University, Villers-lès-Nancy, France / Campus Scientifique, B.P. 239 - 54506 Vandoeuvre-lès-Nancy, France; E-Mails: song@loria.fr, schott@loria.fr

**Keywords:** **S**ensor networks, connectivity, link model, node isolation probability, giant component, critical node density

## Abstract

Connectivity is a fundamental issue in research on wireless sensor networks. However, unreliable and asymmetric links have a great impact on the global quality of connectivity (QoC). By assuming the deployment of nodes a homogeneous Poisson point process and eliminating the border effect, this paper derives an explicit expression of node non-isolation probability as the upper bound of one-connectivity, based on an analytical link model which incorporates important parameters such as path loss exponent, shadowing variance of channel, modulation, encoding method etc. The derivation has built a bridge over the local link property and the global network connectivity, which makes it clear to see how various parameter impact the QoC. Numerical results obtained further confirm the analysis and can be used as reference for practical design and simulation of wireless ad hoc and sensor networks. Besides, we find giant component size a good relaxed measure of connectivity in some applications that do not require full connectivity.

## 1. Introduction and Motivation

Considerable studies have discussed the issues such as the capacity [1] and multi-hop routing [2-4] in wireless *ad hoc* and sensor networks, among which connectivity is a fundamental property to be preserved and also provides design reference for upper-layer protocols. For example, we have to decide a minimum node density to ensure global connectivity and provide route diversity in routing decisions; when we do node sleep scheduling to save energy, the connectivity of active nodes has to be maintained and satisfy the application's QoS requirement.

Pioneering works [5-8] dealing with network connectivity are mostly based on a simplistic Boolean model with the assumption that two nodes are connected if and only if their distance is less than a deterministic transmission radius. Unfortunately, the real low-power wireless links are unreliable and asymmetric, suffering from severe propagation impairments such as path loss, multi-path fading and shadowing, which have great impact on the global QoC. A few papers have dealt with connectivity of *ad hoc* networks in the presence of channel randomness, fading and shadowing [9-11]. Important to our work is the contribution in [9], studying the node isolation probability, from which, under the assumption of dense networks, an approximation of one-connectivity probability can be obtained. In our paper, we adopt an analytical link model which best matches Mica2 Mote's [12] real link state. Through an in-depth derivation, local property of individual links is connected to the global network connectivity. We have shown through both analysis and simulation how various parameters impact the QoC in wireless sensor networks.

The rest of the paper is organized as follows. Section 2 provides a short overview of both wireless link model and network connectivity issue. The detailed analysis of node isolation probability under an analytical link model is presented in Section 3. In Section 4 we do extensive simulations to evaluate the analysis in Section 3 and also discuss the impact of various parameters on the global QoC. Finally we conclude the paper in Section 5 and point out the future direction.

## 2. Related Work

### 2.1. Overview of Wireless Radio Link Models

**Figure 1.**
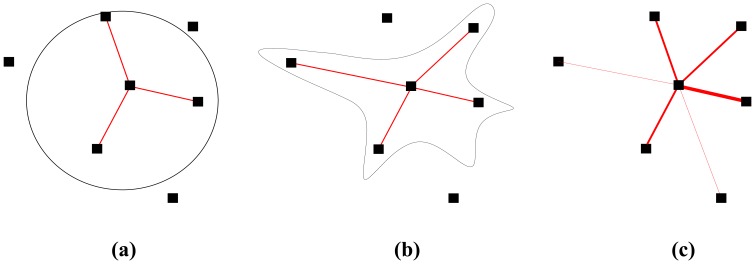
Different kinds link models. **(a)** Boolean disk model. **(b)** Shadowing model. **(c)** Probabilistic model.

A lot of existing works are based on the Boolean disk model, as illustrated in Figure 1(a). Two nodes are either connected or disconnected depending on the transmitter-receiver distance and the disk radius. Recent works [2, 13] argue that the real characteristics of low-power wireless links differ greatly from the ideal model especially in their unreliability and asymmetry. Some studies have improved the disk model and consider the shadowing effect. The radiation pattern is squeezed and stretched as shown in Figure 1(b). It is proved in [14] that irregular radio patterns can achieve connectivity more easily if they can maintain an average number of functioning connections. The two models above are basically deterministic either with assured transmission or assured probability of transmission within the communication range. However, several studies [2, 13, 15-19] have pointed out that the communication range of a wireless node cannot be specified and they propose to use probabilistic link model, as shown in Figure 1(c) (the sickness of the line represents the probability of connection), instead of deterministic model. It is found that the successful transmission probability at a given distance *s*, namely *P*(*s*), is a non-monotonically decreasing function to *s*. *P* relies not only on the distance *s* between two nodes but various parameters such as the channel parameters like the path loss exponent, the shadowing variance and the degree of irregularity (DOI) [16], the radio parameters including modulation, encoding, output power, receiver noise and frame size.

The third link model discussed above is supported by many experimental studies and significantly affects networks behavior. Woo *et al.* have identified in [2] the existence of three distinct reception regions in wireless links: connected, transitional and disconnected. The transitional region is relatively large in size and is characterized by high variance in reception rates and asymmetric connectivity. In a typical sensor networks, a large number of links (even higher than 50%) can be unreliable because of the transitional region. Ganesan *et al.* [17] provide a wealth of empirical data from studies of large scale, dense wireless network, which demonstrate that even a simple flooding algorithm entails complex behavior under unreliable links. Zhou *et al.* [16] find that radio irregularity has a significant impact on the routing protocols in wireless sensor networks, especially location-based routing, such as geographic forwarding. All these research results lead us to stress the need for realistic link models for wireless sensor networks.

Several recent studies have proposed communication models based on empirical data and analyzed related phenomena for more accurate evaluation of upper-layer protocols. Woo *et al.* [2] present a simple synthetic link model to generate data under specific radio and environment based on an assumption of Gaussian distribution of the packet reception rate for given transmitter-receiver distance. These synthetic traces are used for simulation of passive link estimators that snoop traffic over the channel and estimate link qualities. Cerpa *et al.* [15] study the relationships between location and communication properties using non-parametric statistical techniques. They provide a probability density function that completely characterizes the relationship and develop a series wireless network models that produce networks of arbitrary size with empirical observed properties. The accuracy of their models is evaluated through a set of communication tasks like connectivity maintenance and routing. Lal *et al.* [18] propose link inefficiency as a cost metric based on detailed observations of link quality variation and explore how link inefficiency can be measured in energy efficient way. They find that only a few measurements of the channel are sufficient to obtain a good estimate of the cost metric. Leskovec *et al.* [19] concentrate on estimating the link quality between pairs of sensors. Received signal strength is chosen as the link quality indicator. They use dimensionality reduction technique such as SVM to understand the topology of the network and identify potential bottleneck of the network. Zhou *et al.* [16] establish a radio model called the Radio Irregular Model (RIM) with empirical data obtained from the Mica2 platform. RIM takes into account both the non-isotropic properties of the propagation media and the heterogeneous properties of devices. With this model, they find solutions to improve the communication performance in the presence of radio irregularity.

Most important to our work is the analysis of transitional region in [13]. Different from empirical models which require specific radio and environment conditions, the analytical model in [13] has a general methodology and can be used for a number of different configurations and hardware designs. The incorporation of different parameters in the link model greatly facilitates our analysis of network connectivity in presence of unreliable and asymmetric links. Detailed description of the link model we use can be found in Section 3.3.

### 2.2. Connectivity of Wireless Networks: State of the Art

One of the first papers on connectivity in wireless multi-hop networks was [20], which investigated how far a node's message percolates for Poisson distributed nodes on an infinitely large area. More recently, Gupta and Kumar [5] performed a fundamental study on the connectivity of uniform distributed nodes in the asymptotic case. They have shown that when the covered area of each node equals to (log*N*+c(*N*))/*N*, where *N* is the number of nodes in the unit disk and $\underset{N\to +\infty }{\lim}\inf\;c\left(N\right)=+\infty$, the resulting wireless network is asymptotically connected with probability one if *c*(*N*) → +∞. Afterwards, various methods and theories have been applied to the study of connectivity. The model of continuum percolation with the Poisson Boolean model is commonly used to study the phase transition phenomenon in wireless connectivity [14, 20-22]. Considering nodes with identical range distributed in an infinite two-dimensional plane according to Poisson point process, there is critical node density, above which there will be an infinite cluster almost surely (it is commonly abbreviated to a.s. and means the event happens with probability 1 if the plane is infinite), below which there will be bounded clusters a.s.

Noisy links are introduced in [23] to study the impact of interference on the connectivity of ad hoc networks using percolation theory. It is found that there is a critical noise coefficient value above which the network is made of disconnected clusters. When the noise coefficient is small enough, the asymptotic connectivity can be achieved. Indubitably, whether two nodes can communicate with each other at any given distance and any moment also depends on the interference condition, which is caused by simultaneous communication between other nodes in the range. Due to interference, communication between two connected nodes may drop to lower bit rate or even become impossible at certain time. However, in our study we regard this case as a medium access issue instead of connectivity problem. In other words, when the link failure is caused by interference or collision, we say that the network MAC is not effective and the link capacity is reduced, instead of saying that the connectivity between two nodes is of lower degree. In fact, a simple collision free MAC protocol, such as TDMA scheme, can help achieve a better connectivity [23].

Although geometric random graph and percolation theory applied to the study of wireless connectivity shows great effectiveness, their basis is a deterministic radio link model, which reduces their charm in real environment. To date, several papers have investigated the connectivity under realistic radio channel model. Fading and shadowing effects are considered. Miorand *et al.* [24] analyze connectivity issues in one-dimensional ad hoc networks using queueing theoretical approach. They use *GI*/*G*/ ^∞^ queue model (In *GI*/*G*/ ^∞^ queue, the arrival process follows general independent distribution and the service process is general random process, which corresponds to general transmission range distribution and general node placement statistics.) to study broadcast percolation problem with general node placement with fading channel. In [9], it is found that when shadowing gets more severe, the link probability at short distances reduces, while increases at large distances. Longdistance connectivity probability will affect the global network connectivity and the routing performance, similar to the small world [25] networks extended with a few long links. It seems that shadowing fading improves global connectivity. However in our study, we find that the effect has been counteracted with the increase in asymmetric links and thus lead to limited improvement of global connectivity. Our work takes into account of more realistic scenarios and builds a bridge connecting local link property and global connectivity behavior, which makes it clear to see how local link parameters impact network global performance.

## 3. Network Connectivity Analysis

### 3.1. Node Spatial Distribution

Suppose the sensor nodes are scattered from the air and the process resembles Poisson arrival in a service queue. Thus we use a homogeneous Poisson point process with intensity λ to model the spatial distribution of the nodes. This process is defined as follows [26]:

#### Definition 1

Consider a measure space (*X*, Σ, *μ*), where *μ* is a σ-finite measure. A Poisson Point Process (P. P. P.) with intensity λ is a collection of random variables *N*(*A*, *ω*), *A* ∈ Σ, *ω* ∈ Ω, defined on a probability space (Ω, *ℱ*, *P*) such that:

- *N*(·, *ω*) is a counting measure on (*X*, Σ) for each *ω* ∈ Ω.
- *N*(*A*, ·) is the number of nodes in subarea *A* which follows Poisson distribution with mean λ(*A*):
  (1) $$P(N(A)=k)=\frac{\lambda^{k}}{k!}e^{-\lambda (A)}\;,\;\text{all}\;A\in \sum$$
  with an expected value *E*(*N*) = *λ*(*A*) = *ρ*(*A*) ‖*A*‖, *ρ*(*A*) and ‖*A*‖ are node density and size of subarea *A* respectively.
- If *A*_1_, *A*_2_, … are disjoint sets then *N*(*A*_1_), *N*(*A*_2_), … are independent random variables:
  (2) $$P(N(A_{1})=k_{1}\wedge N(A_{2})=k_{2}\wedge \ldots \wedge N(A_{n})=k_{n})=\prod_{i=1}^{n}P(N(A_{i})=k_{i})$$

If *ρ* is constant over the entire infinitely large area, the process is homogeneous. In other words, the outcome of *N* only depends on ‖*A*‖ but not on its particular location or shape. Note that a homogenous Poisson point process can be regarded as a case of uniform distribution of *k* nodes in area *A*, as *k* and ‖*A*‖ tends to infinity but the density *ρ* = *k*/‖*A*‖ remains constant.

### 3.2. Node Non-Isolation Probability

We consider an arbitrary subarea *A* of an infinite plane. In random graph theory [26], connectivity is defined as follows:

#### Definition 2

A graph is said to be *k*-connected if for each pair of nodes there exist at least *k* mutually independent paths connecting them. In other words, a graph is *k*-connected if and only if there is no set of *k*-1 nodes whose removal would disconnect the graph.

Our focus will first be on one-connectivity. Higher orders of connectivity will be discussed in the future work. We are interested in the critical density *ρ*, at which the network *A* is one-connected a.s. In this section, we derive necessary condition for the network to be one-connected. We denote *P*(*I*) as the isolation probability, which is the probability that a randomly chosen node has no neighbors at all. The probability that none of the nodes in *A* is isolated, denoted by *P*(*¯T*) can be the upper bound for the probability that the network is one-connected [9, 27], denoted by *P*(*C*). Thus we have:

(3) $$P(C)\leq P(\bar{I})$$

(4) $$\rho (P(C)=p)\geq \rho (P(\bar{I})=p)$$

Equation (3) is obvious. Equation (4) means under the same network configuration, we need higher node density to reach one-connectivity with probability *p* than to reach node non-isolation with the same probability. We assume the isolation of different nodes in A to be independent events and the number of nodes in *A* is large, i.e. *λ* ≥ 100. The conditional probability of non-isolation is give by:

(5) $$P(\bar{I}|N=k)={\left(1-P(I)\right)}^{k}$$

Thus the unconditional non-isolation probability can be written as:

(6) $$\begin{array}{ll} P(\bar{I}) & =\sum_{k=1}^{\infty }P(\bar{I}|N=k)\cdot P(N=k)\\ & =\sum_{k=1}^{\infty }{\left(1-P(I)\right)}^{k}\cdot \frac{\lambda^{k}}{k!}e^{-\lambda }\\ & =e^{-P(I)\lambda }\sum_{k=1}^{\infty }\frac{{\left((1-P(I))\lambda \right)}^{k}}{k!}e^{-(1-P(I))\lambda }\\ & =e^{-P(I)\lambda } \end{array}$$

Since *P*(*I*) is the probability that a randomly chosen node has no neighbors, we denote *D* to be the number of neighbors of a node, which is also called node degree. Note that *D* also follows Poisson distribution according to (1) and let the expected value of *D* be *D*_0_. Hence *P*(*I*) can be calculated as:

(7) $$P(I)=P(D=0)=e^{-D_{0}}$$

In the following, we calculate the mean node degree *D*_0_. Let *L*(*i*, *j*) denote the event that there is a link between node *i* and node *j*. The distance between the two nodes is known as *s*(*i*, *j*). Given the distance, the probability for *L*(*i*, *j*) is denoted by *P*(*L*|*s*). *D*_0_ can be calculated by integrating *ρP*(*L*|*s*) over the whole plane:

(8) $$D_{0}=\rho \int_{0}^{2\pi }\int_{0}^{\infty }P(s)\cdot \text{sds}\;d\phi =2\pi \rho \int_{0}^{\infty }P(s)\cdot \text{sds}$$

Plugging (7) (8) to (6), we have:

(9) $$P(\bar{I})=\exp(-\rho \left\|A\right\|\exp(-2\pi \rho \xi ))$$

where $\xi =\int_{0}^{\infty }P\left(s\right)\text{sds}$. Let the left hand side of (9) be *p*, then the density *ρ* satisfies the following equation:

(10) $$\rho e^{-2\pi \rho \xi }=\frac{\ln p}{\left\|A\right\|}$$

The solution of the equation is

(11) $$\rho (P(\bar{I})=p)=-\frac{1}{2\pi \xi }\text{Lambertw}(\frac{-2\pi \xi \ln p}{\left\|A\right\|})$$

where Lambertw(*x*) is the solution *w* of equation *w*·exp(*w*)=*x*. Finally, the problem has transformed to the analysis of *ξ* and *P*(*L*|*s*). In other words, the study on global network connectivity has turned into the analysis of local link probability.

### 3.3. Link Probability Analysis

Depending on different radio link model, the link probability *P*(*L*|*s*) is of different values. For Boolean model, either disk or irregular shape as shown in Figure 1(a) and Figure 1(b), *P*(*L*|*s*) is a logical function, equal to either 0 or 1 depending on the communication range. Hence,

(12) $$\xi =\pi r^{2}\rho$$

where *r* is the real communication radius or equivalent radius for Boolean disk model and Boolean shadowing model. For probabilistic model as shown in Figure 1(c), *P*(*L*|*s*) is not deterministic 0 or 1, but defined as the packet reception rate (PRR) ψ over a period of time *τ*. In practice, ψ can be estimated over a moving time window. However, we can use it in simulation as a stationary variable to indicate the probability of instant link success or failure.

Empirical studies have shown that the lognormal shadowing model provides more accurate multipath channel models than other models like Nakagami and Rayleigh models [13]. According to this model, the received power *P_r_* in dB is given by:

(13) $$P_{r}\;(s)=P_{t}-\text{PL}(s_{0})-10\eta \log_{10}(\frac{s}{s_{0}})+N\;(0,\sigma )$$

where *P_t_* is the output power, *η* is the path loss exponent, *N*(0,*σ*) is a Gaussian random variable with mean 0 and variance *σ*^2^, and *PL*(*s*_0_) is the power decay for the reference distance *s*_0_. *σ* can be high in the case of severe signal fluctuations due to irregularities in the surrounding of the receiver and transmitter's antennas.

(13) does not include anisotropic properties of the radio. To incorporate it, we add a coefficient *K_i_* to represent the difference in path loss in different directions. (13) can be modified as :

(14) $$\begin{array}{l} P_{r}\;(s)=P_{t}-\left(\text{PL}(s_{0})+10\eta \log_{10}(\frac{s}{s_{0}})\right)\times K_{i}+N\;(0,\sigma )\\ K_{i}=\{\begin{array}{cc} 1 & \text{if}\;i=0\\ K_{i-1}\pm \text{rand}\cdot \text{DOI} & \text{if}\;0<i<360\;\text{and}\;i\in N \end{array}\\ \text{DOI}=0.01821 \end{array}$$

DOI represents the degree of irregularity. The reference value is suggested by [16] to be 0.01821, which is measured using Mica2 mote. We can generate 360 values for 360 different directions. However, to make the simplicity, we just adopt 4 directions in the simulation.

For a modulation method *M* and encoding method *E*, the PRR ψ is defined as a function of bit-error rate *β*_M_, while *β*_M_ is a function of SNR *γ*. Takeing Manchester encoding for example:

(15) $$\varphi (\gamma )={\left(1-\beta_{M}(\gamma )\right)}^{8(2\;f-h)}$$

where *f* is the frame size (preamble, payload and CRC), *h* is the preamble (both in bytes). The value of *β*_M_ depends on modulation, and more generally on transmitting and receiving techniques (diversity, equalization, etc.). Here we take non-coherent FSK for example, which is the default modulation scheme in Mica2 Mote. Expressions for *β*_M_ is:

(16) $$\beta_{M}=\frac{1}{2}e^{-\frac{\gamma }{2}}\;,\;\gamma =10^{\gamma_{dB}/10}$$

Plugging (16) into (15), we have:

(17) $$\Psi (\gamma )={\left(1-\frac{1}{2}e^{-\frac{\gamma }{2}}\right)}^{8(2\;f-h)}$$

The SNR *γ* can be obtained from (14) and is given by:

(18) $$\gamma_{dB}\;(s)=P_{r}\;(s)-P_{n}$$

where *P_n_* is the noise floor which depends on both the radio and the environment. Considering an ambient temperature of 27°C and no interference signals, the noise floor is −115 dB [13]. Note that *γ* in (18) can also be regarded as a Gaussian random variable *N*(*μ*(*s*),*σ*) with mean *μ*(*s*) and variance *σ*^2^, where *μ*(*s*) is given by:

(19) $$\mu (s)=P_{t}-(\text{PL}(s_{0})+10\eta \log_{10}(\frac{s}{s_{0}}))\cdot K_{i}-P_{n}$$

**Figure 2.**
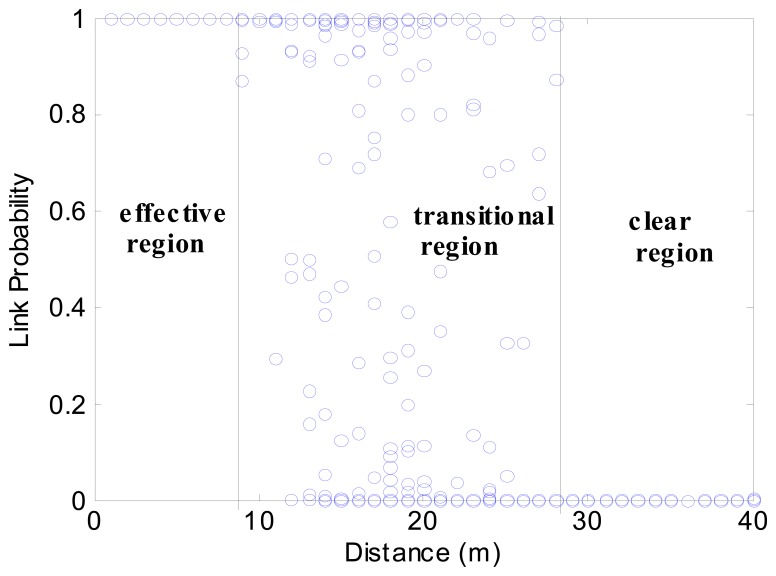
Analytical PRR to distance, obtained from (19) and (17), *P_t_*=-5 dB, *η*=3, *σ*=3.3, *f*=50 Byte, *h*=2 Byte.

According to (19) and (17), given the distance and some related parameters, the PRR can be calculated. Figure 2 shows the analytical model of PRR to distance in one direction. It is clear that there is a large transitional region, in which the PRR is unstable. With the following definitions, we can further outline the transitional region.

#### Definition 3

If the link between two nodes has a high probability (>*p_h_*) of having high PRR (>ξ*_h_*), then the two nodes are strongly connected. The upper bound of the connected range is *s_l_*.

#### Definition 4

If the link between two nodes has a high probability (>*p_h_*) of having low PRR (<ξ*_l_*), then the two nodes are almost disconnected. The lower bound of the disconnected range is *s_h_*.

According to definition 3 and definition 4, the region between *s_l_* and *s_h_* is the transitional region, as illustrated in Figure 2. This agrees with empirical observations in [2, 28]. The existence of this region has been explained in [13], which is the joint effect of non-perfect-threshold receiver, noisy environment and multi-path effects.

**Figure 3.**
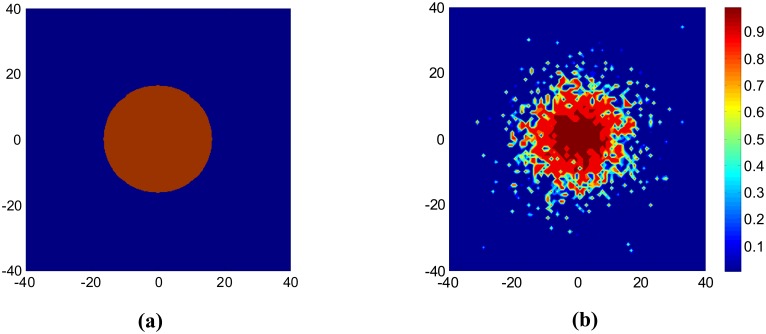
Contour plot for different link models. (a) Boolean disk model**. (b)** Log-normal shadowing based probabilistic model.

With consideration of different pass loss and shadow fading along different direction, we draw contour plot of the reception rate for a node located at the center of the region. Figure 3 shows the difference of the communication area of a Boolean disk model and a shadowing fading model. Figure 3(b) captures the real characteristic of wireless link and shows spatial irregularity as the empirical results in [14].

To calculate *P*(*Ī*), the value of *ξ* is crucial. As depicted in Figure 2, it is difficult to derive a continuous function for *P*(*L*|*s*). Instead, we calculate the expected value of *P*, denoted as *E*(*P*). Thus, *ξ* can be written as:

(20) $$\xi =\int_{0}^{\infty }P\left(L|s\right)\text{sds}\approx \int_{0}^{\infty }\int_{-\infty }^{\infty }\Psi \left(\gamma \right)f\left(\gamma |s\right)\cdot sd\gamma ds$$

where *f*(*γ*|*s*) represents the probability density function of SNR. According to (17), we draw the PRR as a function of *γ*. As the power-law relationship entails a sharp threshold, linear approximation turns available. Ψ(*γ*) can be approximated as the following piecewise linear functions:

(21) $$\begin{array}{c} \Psi \left(\gamma \right)=\{\begin{array}{ll} 0 & \gamma \leq \gamma_{0}\\ k_{\varphi }\gamma +b_{\varphi } & \gamma_{0}<\gamma <\gamma_{1}\\ 1 & \gamma \geq \gamma_{1} \end{array}\\ \begin{array}{cc} k_{\varphi }=\frac{0.9-0.1}{\gamma_{1}-\gamma_{0}}, & b_{\varphi }=\frac{0.1\gamma_{1}-0.9\gamma_{0}}{\gamma_{1}-\gamma_{0}} \end{array}\\ \begin{array}{cc} \gamma_{0}=\Psi^{-1}\left(0.1\right), & \gamma_{1}=\Psi^{-1}\left(0.9\right) \end{array} \end{array}$$

where *γ*_0_ and *γ*_1_ represent the receiver's range of threshold.

Similarly, *f*(*γ*|*s*) can also be evaluated by linear approximation since the interval [*γ*_0_, *γ*_1_] is relatively narrow compared with [*μ*-4σ, *μ*+4σ].

(22) $$\begin{array}{l} f(\gamma |s)=k_{f}\;(s)\gamma +b_{f}\;(s),\gamma \in [\gamma_{0},\gamma_{1}]\\ k_{f}\;(s)=\frac{f\;(\gamma_{1}|s)-f\;(\gamma_{0}|s)}{\gamma_{1}-\gamma_{0}},b_{f}\;(s)=\frac{f\;(\gamma_{0}|s)\gamma_{1}-f\;(\gamma_{1}|s)\gamma_{0}}{\gamma_{1}-\gamma_{0}} \end{array}$$

**Figure 4.**
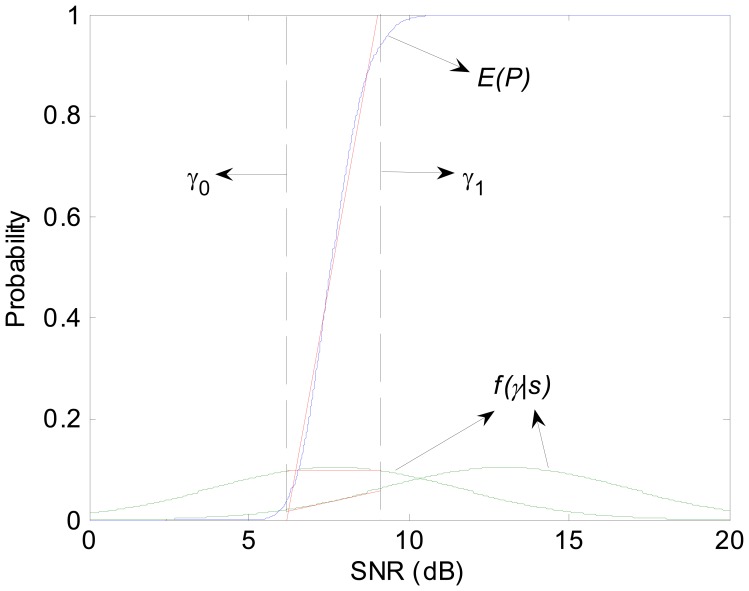
The procedure of linear approximation for Ψ(*γ*) and *f*(*γ*|*s*).

Figure 4 shows the approximation procedure for Ψ(*σ*) and *f*(*y*|*s*). Finally, *ξ* can be approximated by:

(23) $$\begin{array}{ll} \xi & =\int_{0}^{\infty }\int_{-\infty }^{\infty }\Psi \left(\gamma \right)\;f\;\left(\gamma |s\right)\cdot sd\gamma ds\\ & \approx \int_{0}^{\infty }\int_{\gamma_{0}}^{\infty }\Psi \left(\gamma \right)\;f\;\left(\gamma |s\right)\cdot sd\gamma ds\\ & \approx \int_{0}^{\infty }(\int_{\gamma_{0}}^{\gamma_{1}}(k_{\varphi }\gamma +b_{\varphi }\;)(k_{f}\;(s)\gamma +b_{f}\;(s))d\gamma +(1-\Phi (\frac{\gamma_{1}-\mu (s)}{\sigma })))\text{sds}\\ & \approx \int_{0}^{d_{h}}\int_{\gamma_{0}}^{\gamma_{1}}(k_{\varphi }\gamma +b_{\varphi }\;)(k_{f}\;(s)\gamma +b_{f}\;(s))\cdot sd\gamma ds+\int_{0}^{d_{h}}(1-\Phi (\frac{\gamma_{1}-\mu (s)}{\sigma })))\text{sds} \end{array}$$

where Φ(·) is the cdf of SNR and *μ* is given by (19). In fact, the upper bound of the integration can be any value lager than *s_h_*. The approximation deviation by lowering the integration upper bound from ∞ to *s_h_* can be tolerated.

In the following, we will use simulations to verify the theoretical analysis.

## 4. Simulation Results and Discussion

In this section, various simulations are conducted to validate the analytical results and show how different parameters impact the network connectivity. *n* nodes are distributed in a square area *A*, following homogeneous Poisson point process. The transmission power is the same for each node. Analytical results are obtained under the assumption that the plane is infinite, in which there are no border nodes. However in simulation we cannot find such a plane. To approach the theoretical assumption, the best way is to eliminate the border effects. To avoid the border effect, we use a wraparound distance model (also called cyclic distance model) instead of usual Euclidean distance model. A node at the border of *A* is considered as being close to the nodes on the opposite border of *A*. Thus it can establish links via the borderline to these nodes. We perform simulations with the increase of node density *ρ* while changing other related parameters. For each unique setting, we repeat simulation runs independently 500 times and finally evaluate the average performance. In the resulting graph for each run, we check the analytical and real node isolation probability, one-connectivity probability, largest component size and giant component probability.

For different applications, the requirement of QoC can be different. For example, a surveillance sensor system with one sink node requires the network to be one-connected so as to collect data from any node in the network. However to detect or track an intrusion [29, 30], it is required that only the nodes around the intrusion connect to the sink. Therefore it is argued that for some use of sensor networks, one-connectivity is a too stringent condition and giant component is suggested as a measure of connectivity instead. In classical random graph *G*(*n*,*p*) [26], every pair of a set of *n* vertices is chosen to be an edge with probability *p*. The behavior of the size of the largest component in *G* when *p*=*c*/*n* for *c* near 1 receives most interests in the field. For *c*<1 the size of the largest component is *O*(log*n*) a.s.; for *c*=1 the size of the largest component is Θ(*n*^2/3^) a.s.; for *c*>1 the size of the largest component is a.s. Θ(*n*) and the second largest component is a.s. *O*(log*n*). When *c*>1, the largest component, whose order is much larger than any other component, is commonly referred to as the giant component. Such random graphs are fundamental and useful for modeling problems in many applications. Although the link model adopted in our paper is quite different from classical one, we can still use the concept of giant component. In our simulation, if half of the nodes in the network are connected, a giant component is formed. This definition actually agrees with the third case (*c*>1) of the above description in random graph theory. It should be note that we use the following notation in the analysis above: $f=O\left(g\right)\;\text{if}\;{\lim\sup}_{n\to \infty }\frac{f\left(n\right)}{g\left(n\right)}<+\infty$; *f*=Θ(*g*) if *f*=*O*(*g*) and *g*=*O*(*f*).

To determine the probability of giant component and one-connectivity of a wireless *ad hoc* network graph, a simple flooding algorithm can be adopted. A random node is tagged at first, and then its neighbors are tagged, which subsequently continue to tag their untagged neighbors, until the corresponding cluster is completely formed. The procedure is repeated for all the untagged nodes until all the nodes in the graph are tagged. By definition, if the size of the largest cluster found is larger than ½*n*, the giant component exists. For an *ad hoc* sensor network to be one-connected, the size of the largest component should equal to the total number of nodes in the network. Theoretically, there is another way to obtain the ratio of one-connectivity network by using Laplacian matrix. Let *G*=(*V*, *E*) be a graph on vertex with vertices *V* and edges *E*. The Laplacian matrix of a graph *G* with *N* nodes is an N×N matrix *L*(*G*)=*D*(*G*)-*A*(*G*), where *D*(*G*) is the diagonal matrix of vertex degrees, *D*(*G*)=diag(*d*(*ν_i_*)), *ν_i_*∈*V*; *A*(*G*) is the adjacent matrix of *G*. The eigenvalues of *L*(*G*) are called the Laplacian eigenvalues, which are all real and nonnegative. The set of all *N* Laplacian eigenvalues λ_1_≥λ_2_≥…≥λ_N-1_≥0=λ_N_ is called the Laplacian spectrum of a graph *G*.

### Theorem 1

If the graph *G* has *C* connected components, then *L*(*G*) has exactly *C* zero eigenvalues (other eigenvalues are positive). Based on Theorem 1, the graph is one-connected if *L*(*G*) possesses only one zero eigenvalue. The probability for one-connectivity is estimated from a representative sample of *ad hoc* sensor networks' connectivity graph as the ratio between one-connected graph and all sample graphs.

**Figure 5.**
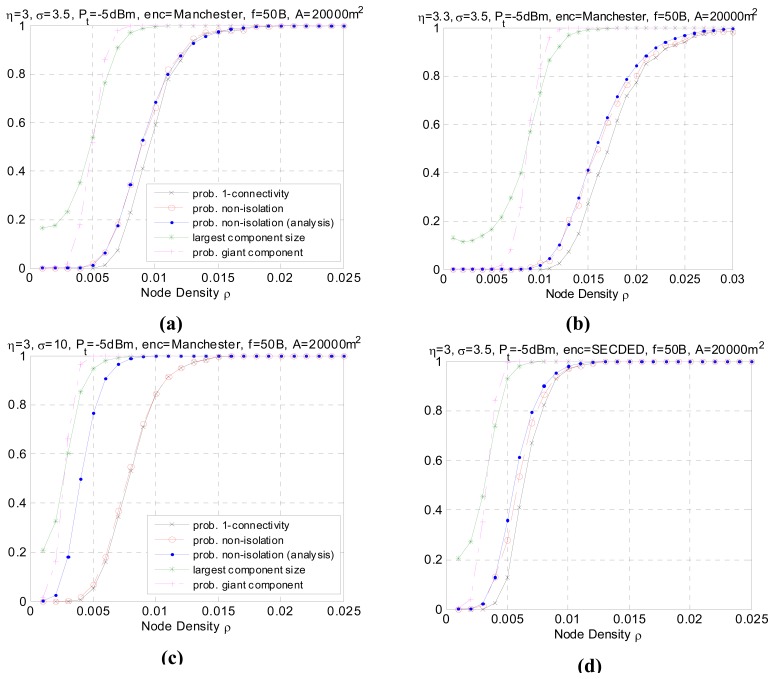

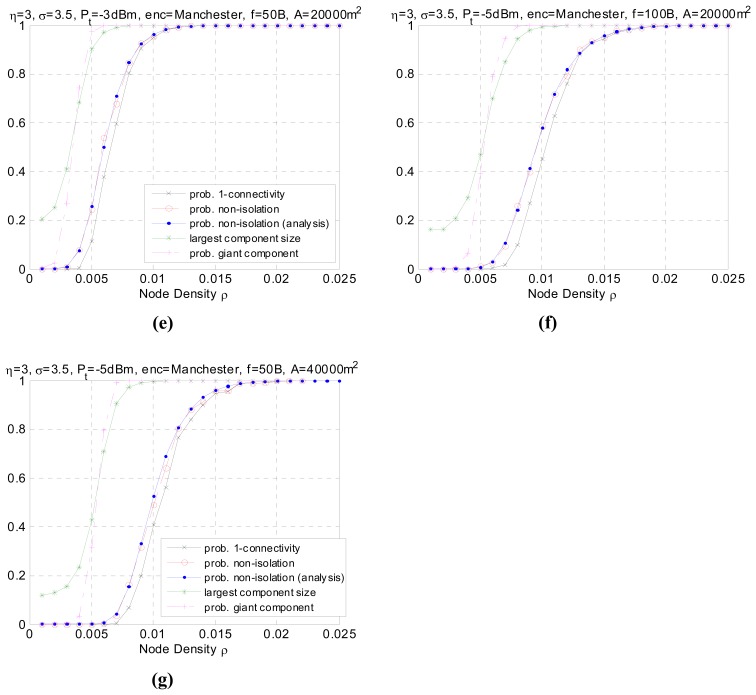
Simulated results for different settings. **(a)** Benchmark. **(b)** Change path loss exponent *η*. **(c)** Changing shadowing variance *σ*. **(d)** Change encoding method *E*. **(e)** Change transmission power *P_t_*. **(f)** Change frame size *f*. **(g)** Changing area size ‖*A*‖.

Figure 5 shows the simulated results. Each subplot corresponds to a different configuration. Figure 5(a) serves as a benchmark for comparison. The critical node densities in different scenarios are reported in Table 1. Through comparing and analyzing the 7 groups of simulated data, we draw the following conclusions and give the explanations.

- In all simulation cases, as the node density increases, the network graph becomes denser and the transition from low connectivity to nearly full connected or appearance of giant component is quite sharp over a small range of *ρ*. The phenomenon consists with the results in [22] which uses the theory of continuum percolation. No matter what kind of radio link model adopted, either Boolean disk model or more realistic model with fading and shadowing, the phenomenon of phase transition exists, which gives us a tool for analyzing and determining resource efficient regime of operation for wireless sensor networks. For example, following the settings in Figure 5(a), it tells us that for the nodes with identical transmission power, distributed in an area of 20000 m^2^ according to homogeneous Poisson process, the node density must be higher than 0.007 to form giant component and higher than 0.0125 to reach one-connectivity. The density threshold is an energy- efficient point of operation, in that to the left of this threshold the network is disconnected with high probability, and to the right of this threshold, additional energy expenditure results in a negligible increase in the high probability of connectivity.
- In all simulation cases, the non-isolation probability serves as the upper bound for probability of one-connectivity. Generally, the difference between the two probabilities is non-negligible. However as node density increases, the two probabilities converge to 1. This result agrees with inequality (3). With respect to critical node density, this means that
  (24) $$\rho \left(P\left(C\right)=p\right)=\rho \left(P\left(\bar{I}\right)=p\right)+\delta ,\;\delta >0.\;\text{As}\;p\to 1,\;\delta \to 0.$$
  For *p*=90%, the critical node densities for each setting are listed in Table 1. It is observed that when the shadowing effect σ is of a large value (*σ*=10), the difference *δ* becomes very small over the whole node density range. This can be explained by the fact that as *σ* increases, the increase in long links and the decrease in short links have reduced the correlation between links. As a result, the geometric random graph behavior approaches generic random graph behavior and the probability of one-connectivity approaches the probability of non-isolation.
- It should be noted that, the analytical performance of non-isolation probability closely matches the real non-isolation probability in different settings except when *σ* is large (*σ*=10), as shown in Figure 5(c). This is also reflected in Table 1 with minor difference between the simulating and analytical critical density for *P*(*Ī*) except when *σ*=10. In some related papers [10-11], it is argued that a large value of *σ* helps the network to be connected because the number of added long links is larger than the number of removed short links. However, it should be note that the network also suffers severe link asymmetry as the shadowing variance increases. The analytical approach has underestimated the asymmetry problem, which leads to an overestimate of network connectivity. In practical, the analytical values have to be calibrated with certain asymmetry coefficient to match the simulated data. Besides, in practice, some realistic issue like antenna diversity, battery power difference etc. will also cause link asymmetry and is more complicated to simulate. We will leave it as future work.
- Comparing Figure 5(b) with Figure 5(a), we can see that the path loss decreases the connectivity of the network. The reason is obvious since the higher *η*, the faster the decay of the signal strength, resulting in a shortened transmission range. The simulated result agrees with the analytical one. Similar is Figure 5(e) and 5(g) compared with Figure 5(a). As the transmission power increases, the average transmission range also increases accordingly, thus a lower node density is sufficient to make the network connected with the same high probability, as shown in Table 1. The giant component probability shows consistent tendency.
- Figure 5(d) shows the impact of a different encoding scheme. The connectivity is better using SECDED encoding than Manchester encoding. This result is due to the error correction capabilities of SECDED, which comes at a cost of energy efficiency (encoding ratio 1:3) while Manchester does not provide error correction and the encoding ratio is 1:2. Simulated results for different encoding schemes agree with the expected analytical behavior.
- Figure 5(f) shows the connectivity performance as the packet size is doubled. It can be observed that the performance merges to 1 at a slightly higher node density. The impact of packet size on the global connectivity is not obvious compared to other parameters.
- The impact of area size ‖*A*‖ on the connectivity is straightforward. As the sub-area size gets larger, to reach the global connectivity requires higher node density. The quantity can be estimated via Equation (10). However, to form a giant component, the probability has not been affected by the area size ‖*A*‖.

In summary, a higher transmission power, larger shadowing variance and more complicated encoding method can improve the network connectivity. However, it is energy inefficient to sacrifice the transmission power for better QoC unconditionally; higher shadowing variance *σ*^2^ can increase the chance of link asymmetry and typically comes with a higher path loss exponent *η*; complicated encoding method consumes more system resource (energy, memory space etc.) and takes more time. With reference to the improvement space of each parameter, one should better balance the connectivity performance and the sacrifices of resource and energy. Other techniques such as collaborative radio [31] and multiple antennas [10], although effective in theory to improve global connectivity, need more detailed infrastructure design and proof of their effectiveness in practice. In all simulation cases, we find that the giant component probability converges to 1 much faster than one-connectivity. In some applications, a few isolated nodes or small clusters outside the giant component do not change the overall performance. In that case, a lower node density is sufficient for operation. Besides, the largest component size is also an alternative of connectivity measurement. It not only provides information about the network is one-connected or not, but also the fraction of the connected part. Thus a random size of largest component can be designated satisfying different requirement of QoC.

## 5. Conclusions and Future Work

In this paper, we have presented an analytical procedure of calculating the node non-isolation probability based on a generalized radio link model in wireless sensor networks. The non-isolation probability is the upper bound of one-connectivity probability. We use a combination of analytical and simulation-based methods to study the impact of various parameters on the connectivity behavior. The results can be applied for practical design and simulation in wireless *ad hoc* and sensor networks. For example, given the environmental and node configuration, we can determine the minimum density needed to achieve a one-connected network or less strictly, form a giant component, according to different requirement of QoC.

Several issues remain for future work in this area. First, this paper only focus on the connectivity in the spatial domain, it is also interesting to study the temporal dynamics of wireless connectivity. For more challenging dynamic environment, statistics of time-varying links are required; second, the giant component size needs further analytical study. It is important if an explicit expression could be derived for the giant component probability or largest component size; third, this paper assumes the node distribution follows homogeneous Poisson point process. However, in many real-world scenarios, the nodes are in an inhomogeneous distribution. Bettstetter *et al.* in [32] has proposed an algorithm to create random inhomogeneous distribution. We believe the more generalized distribution model will lead to more convinced analytical and numerical results. Fourth, it will be interesting if we incorporate the context of MAC layer, say IEEE 802.15.4, so that connectivity is more information based than pure physical link. Finally, *k*-connectivity is a more generalized measure of global connectivity and provides more redundancy for routing choice. In other words, one-connectivity is just a special case for k-connectivity. Some analysis has been conducted in [33], but still need further investigation. The procedure may find some similarities, but the result is of greater generality.

## Figures and Tables

**Table 1.** Critical density for giant component, one-connectivity and non-isolation under different settings, comparison of analytical and simulation results.

**Settings**	***ρ*(*P*(*G*)=90±.05%) (m^-2^) simulation**	***ρ*(*P*(*C*)=90±.05%) (m^-2^) simulation**	***ρ*(*P*(*Ī*)=90±.05%) (m^-2^) simulation**	***ρ*(*P*(*Ī*)=90±.05%) (m^-2^) analysis**
(a)	7.00 · 10^-3^	1.25 · 10^-2^	1.22 · 10^-3^	1.25 · 10^-3^
(b)	1.15 · 10^-3^	2.27 · 10^-2^	2.21 · 10^-3^	2.15 · 10^-3^
(c)	4.50 · 10^-3^	1.07 · 10^-2^	1.07 · 10^-3^	6.00 · 10^-3^
(d)	4.80 · 10^-3^	8.75 · 10^-3^	8.50 · 10^-3^	8.00 · 10^-3^
(e)	5.00 · 10^-3^	9.00 · 10^-3^	8.70 · 10^-3^	8.70 · 10^-3^
(f)	7.50 · 10^-3^	1.33 · 10^-2^	1.30 · 10^-3^	1.32 · 10^-3^
(g)	7.00 · 10^-3^	1.40 · 10^-2^	1.35 · 10^-3^	1.32 · 10^-3^
